# Perspectives of refugee parents and unaccompanied minors on initial health assessment and access to care

**DOI:** 10.1007/s00431-024-05523-5

**Published:** 2024-04-09

**Authors:** Albertine Baauw, Chanine F. S. Brouwers, Sogol Fathi Afshar, Johannes B. van Goudoever, Mai J. M. Chinapaw, Mariëtte H. H. Hoogsteder

**Affiliations:** 1 Amsterdam UMC, Department of Public and Occupational Health, Amsterdam Public Health Research Institute, Vrije Universiteit Amsterdam, Boelelaan 1117, Amsterdam, The Netherlands; 2 Amsterdam UMC, Amsterdam Reproduction and Development Institute, University of Amsterdam, Emma Children’s Hospital, Meibergdreef 9, Amsterdam, The Netherlands; 3 Training Institute Global Health and Tropical Medicine, Dutch Society of Global Health and Tropical Medicine, Utrecht, The Netherlands; 4 Department Health and Society, Wageningen University and Research, Wageningen, The Netherlands

**Keywords:** Refugee, Asylum-seeking parent, Unaccompanied minor, Migrant health, Initial health assessment, Access to care, Risk-based screening

## Abstract

To explore the needs, expectations, and experiences of asylum-seeking parents and unaccompanied minors under the age of 18 years on the initial health assessment for children and adolescents and access to care upon entry in the Netherlands, We conducted five semi-structured focus group discussions with asylum-seeking parents and unaccompanied minors, from Syria, Eritrea, Afghanistan, and other Middle-East and African countries, supported by professional interpreters. To triangulate findings, semi-structured interviews with health care professionals involved in care for refugee children were conducted. Transcripts of focus group discussions were inductively and deductively coded and content analyzed; transcripts of interviews were deductively coded and content analyzed. In total, 31 asylum-seeking participants: 23 parents of 101 children (between 0 and 18 years old), 8 unaccompanied minors (between 15 and 17 years), and 6 healthcare professionals participated. Parents and minors expressed that upon entry, their needs were met for vaccinations, but not for screening or care for physical and mental health problems. Parents, minors, and health professionals emphasized the necessity of appropriate information and education about health, diseases, and the health system. Cultural change was mentioned as stressful for the parent–child interaction and parental well-being.

*     Conclusion*: The perspectives of refugee parents and unaccompanied minors revealed opportunities to improve the experience of and access to health care of refugees entering the Netherlands, especially risk-specific screening and more adequate education about health, diseases, and the Dutch health care system.

**Table:** 

**What is Known:** *• Refugees have specific health needs due to pre-flight, flight, and resettlement conditions. Health assessment upon entry was non-obligatory in the Netherlands, except for the tuberculosis screening. Health needs were not always met, and refugees experienced barriers in access to care.*	
**What is New:** *• The initial health assessment met the needs concerning vaccinations but mismatched the needs regarding physical and mental health assessment. Screening for specific risk-related diseases and mental health could enable refugee parents and minors to engage better with the health system.*

**Supplementary Information:**

The online version contains supplementary material available at 10.1007/s00431-024-05523-5.

## Introduction

The number of people forcibly displaced from their homes increased considerably over the past years worldwide. Among the nearly 90 million of them, about 27 million were refugees in 2021. Minors under the age of 18 years, making up about a third of the world population, constitute about half of the refugee population [1]. In 2021, over 120,000 refugees arrived in Europe, despite the COVID-19 pandemic. In the Netherlands, the majority of refugees enter through an asylum procedure or family reunification afterward; new asylum requests from 2015 to 2021 counted 237,000 applications, of whom nearly 42,000 children under the age of 18 (Appendix I [2, 3]).

More than 12,000 came unaccompanied by a parent. Asylum-seekers from Syria were the largest group, followed by people from Eritrea. Other important countries of origin were Nigeria, Iran, Iraq, and Afghanistan [4].

Refugees often have complex health care needs, ranging from infectious and chronic diseases to mental problems or disorders [5–7]. Refugee children have an increased risk for a variety of conditions compared to children born in the Netherlands, including anemia, genetic disorders of the red blood cells, infectious diseases, growth and nutrition disorders, incomplete vaccination status, and psychosocial problems [8]. Unfavorable conditions in their home countries, the flight and migration itself, the stress of living in unfamiliar surroundings, and the uncertainty about their asylum-seeking status all contribute to increased risks and vulnerability of mental and physical health problems [9–11].

In the Netherlands, Youth Health Care monitors the health status of all children, through antenatal and neonatal screening and follow-up during childhood and adolescence. Youth Health Care also conducts the initial health assessment of asylum-seeking children and adolescents arriving in the Netherlands, taking place during their stay in reception centers. This initial health assessment is non-mandatory and consists of an intake with anamnesis, a physical examination of growth and development, and an evaluation of the health status and well-being of the children [12]. An inventory of the vaccination status is made, and necessary additional vaccinations are provided [13]. Screening for tuberculosis is provided upon entry, for detecting pulmonary tuberculosis in people from high prevalence countries. When required, children are referred to specialist care services. This initial health assessment does not include laboratory tests or standardized mental health screening.

The initial health assessment is crucial for access to care because it focuses on the early detection of health problems and needs and initiates referrals to health care services. Levesque et al. defines access to health care as “the opportunity to have health care needs fulfilled” [14]. Perspectives of asylum-seekers on their health, the assessment, and the care provided in their reception country are of key importance to understand access and match their needs. However, their perspectives have scarcely been studied. The first European study investigating the perspective of asylum-seeking and refugee caregivers was about the quality of care provided in a pediatric tertiary hospital. It described a mismatch of personal competencies and external challenges such as communication barriers and unfamiliarity with new health concepts [15]. Other studies about parental perspectives identified important barriers for accessing health care and unmet health needs [16].

The aim of the current study was to better understand how asylum-seeking parents of children and unaccompanied minors experience the initial health assessment and access to care after arriving in the Netherlands.

## Methods

### Design, context, and ethics

We designed a qualitative study with focus group discussions (FGD) and semi-structured interviews. Focus groups consisted of asylum-seeking parents and unaccompanied minors with rejected asylum requests. Interviews were conducted with health care professionals working in reception centers and with other refugees than our study population. From 50 reception centers in the Netherlands in 2018, we approached one center in Arnhem in which many families reside, hosting on average about 400 people, among which are 100 children. The inhabitants were asylum-seekers waiting for their asylum decision and refugees with a residence status, waiting for a regular house or apartment. Focus group discussions (FGD) with parents of children were held in this center. A nearby small-scale housing facility in Arnhem for unaccompanied minors (UMs) with rejected asylum request was approached to recruit minors. The personnel of the reception center were informed in a training about the study, the independency of the researchers, and the informed consent procedure to ensure voluntary participation in the recruitment, transparency, and confidentiality.

### Recruitment and informed consent

Parents and UMs were recruited in collaboration with employees from the National Central Organ of Asylum-seekers (COA), working in the Arnhem Center or minor housing facility. The employees orally invited parents and UMs through purposive sampling to voluntarily participate in a group discussion about health assessment and access. They distributed a study flyer in five languages to ensure representation from the main regions of origin. Researchers provided written and oral information about the study to the managers of the locations to the UM guardians who had custody and to the participants themselves for recruitment. At the start of each FGD, we gave the informed consent forms in the specific language at hand to the participants and discussed—via an interpreter—information and consent with them. Researchers answered questions of participants, and, after that, participants gave written consent. UMs’ guardians were asked permission to conduct a FGD with minors; they gave written consent after oral assent by the minors. Health professionals were recruited with the snowball technique, starting with professionals in the network of the first authors.

In the report of this study, we followed the COREQ guidelines [17].

### Data collection, language, and interpretation

At each FGD, a professional interpreter, recruited through a licensed bureau for interpreters, was present to translate between Dutch and the mother tongue of the participants (two FGDs in Arabic, one Farsi, one Dari, and one Tigrinya). The interpreters were briefed before to explain the aim of the FGD and the most important concepts. The questions were asked in Dutch and translated in the language of the participants, and the answers were translated back into Dutch. The transcripts were analyzed and coded in English.

### Analysis

**Fig. 1:**
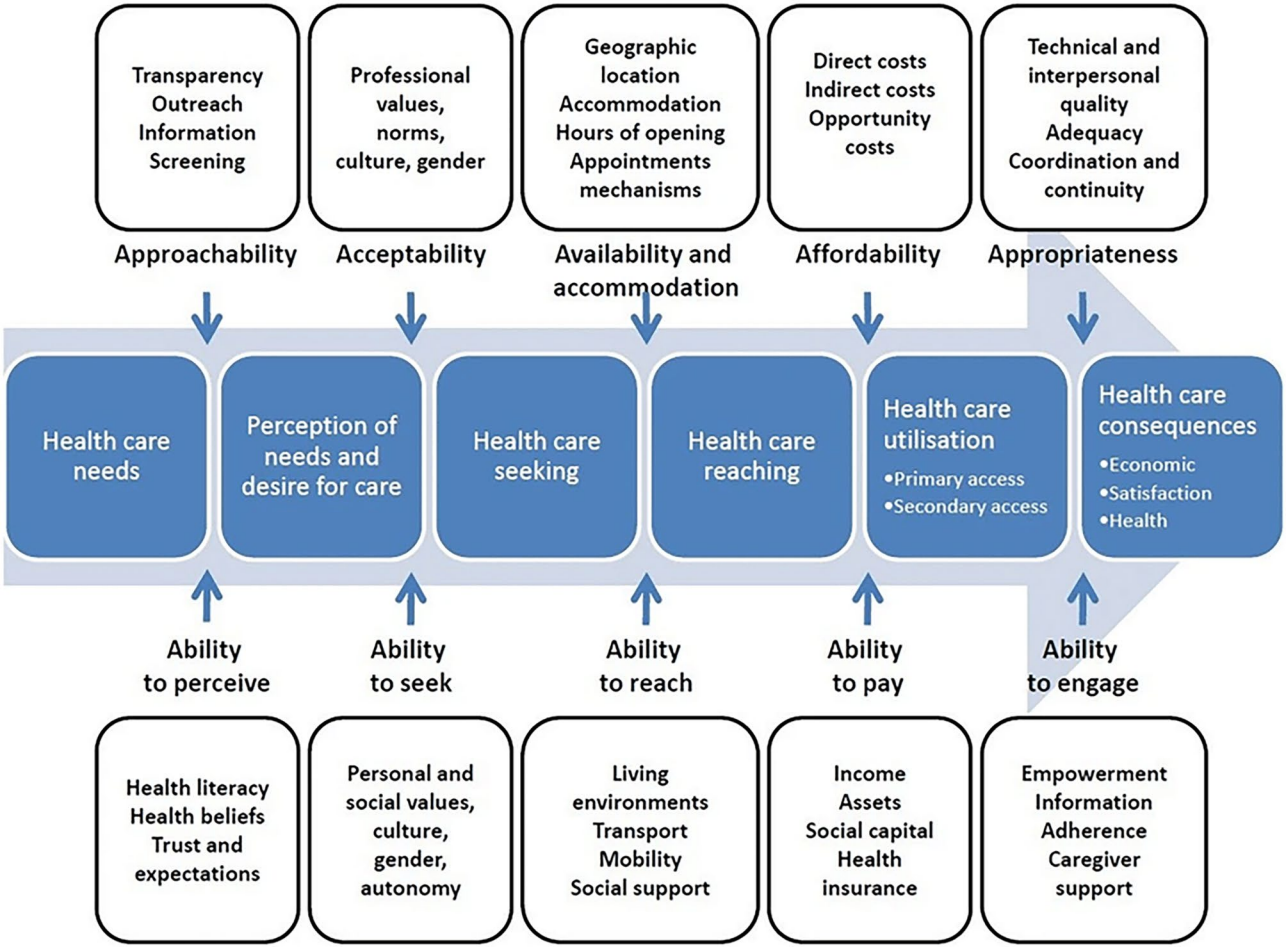
Note: A conceptual framework of access to health care (Levesque et al. [14]). Reprinted from “Patient-centred access to health care: conceptualising access at the interface of health systems and populations” by J-F. Levesque, M.F. Harris, and G. Russell, 2013, International Journal for Equity in Health, 12(1), p. 5.]

The audio-recorded FGDs and interviews were transcribed using verbatim style. The transcripts were coded with ATLAS.ti 7 software (ATLAS.ti GmbH, Berlin, Germany). A combination of a deductive and inductive approach was used in the analysis. Each transcript was read several times in order to gain familiarity with the data. A deductive approach was used for all fragments concerning access to health care by health care professionals based on the five access dimensions (approachability, acceptability, availability, affordability, and appropriateness) and their corresponding patient abilities (ability to perceive, to seek, to reach, to pay, and to engage) by Levesque [14] (see Fig. 1). Next, open coding was used, and the subsequent codes resulted in a coding scheme. Then, axial coding was used for all fragments concerning experiences and needs of the study group on the initial health assessment. Data analysis was a circular process of going back and forth adding new codes or labels where necessary [18]. Final themes were created based on the prevalence of certain codes and their interrelation, or the salience of a theme in relation to the research questions. Appendix II contains the final coding scheme. Two researchers (CB, SFA) coded the transcriptions. Two others (AB, MH) double-coded a selection of the transcripts. Differences were discussed until a consensus was reached.

## Results

A total of 31 participants were involved in the FGDs; 23 parents (12 mothers, 11 fathers) of one to twelve children per parent—but not all children lived in the center or in the Netherlands and 8 unaccompanied minors with a negative asylum decision (male, aged 15 to 17 years). The parents were either in the asylum process or were recently granted residency in the Netherlands but still living in the center waiting for accommodation elsewhere. Participants came from nine countries, mostly from Syria, Eritrea, or Afghanistan (Table 1).

**Table 1:** Characteristics of participants in focus group discussions

	**Parents (*n* = 23)**	**UMs (*n* = 8)**
Gender		
Male	11	8
Female	12	
Age in years		
15		1
16		5
17		2
Country of origin		
Afghanistan	4	
Eritrea		5
Guinee		1
Iran	2	
Iraq	2	
Jordan	1	
Kuwait	2	
Sudan		2
Syria	11	
Number of children per parent		
1–2	3	
3	5	
4	9	
5–6	2	
7–12	4	

Focus group sizes varied between five and eight participants.

Six health care professionals were interviewed (5 female; 4 face-to-face, and 2 by telephone): three doctors (GP, Pediatrician, Youth Health physician), one nurse, one manager, and one policy advisor. The doctors and nurse had daily or weekly consultations with refugees, the two other professionals worked exclusively in health care for refugees (Table 2).

**Table 2:** Characteristics of interviewed health professionals

Code	Gender	Type of interview	Professional position in refugee health
HP1	Female	Face-to-face	Pediatric Hematologist^b^
HP2	Female	Face-to-face	General Practitioner GZA^b^
HP3.1^a^	Female	Face-to-face	Manager YHC^c^
HP3.2^a^	Female	Face-to-face	Strategic Advisor YHC^c^
HP4	Female	Telephone	Nurse YHC (children 4–18 years)^b^
HP5	Male	Telephone	Doctor YHC (children 0–18 years)^b^

^a^double-interview with two professionals

^b^seeing refugees daily or weekly

^c^working for refugees

### Initial health assessment

In general, parents and unaccompanied minors did not understand that they had received an initial health assessment, with the exception of being asked about and possibly receiving vaccinations. Vaccinations were well received, both by parents and adolescents. Some minors confessed they were reluctant to go for future vaccinations or what they called check-ups, for example, because they were afraid of injections or perceived they did not need them (“I feel healthy”). Some were unable to find the location or felt barriers to see a doctor (Table 3, quote 7). (See Appendix IV for all characteristics of the participants).

**Table 3:** The initial health assessment: quotes illustrating experiences, needs, and expectations of asylum-seeking parents and UMs

Respondent	Phrase
1	People come from war zones and during the war they contracted diseases and certain conditions. Due to the war we did not have access to health care or proper treatment….. So we expect an extra check-up. That would be good. If you have psychological issues, you need to see a doctor, is something bothering you. Then people do not feel excluded. You feel acknowledged, both physically and mentally… Syrian father of 4 children
2	Anemia, that happened a lot last years. There was a shortage of food, with the war you know. The last three years in Syria were bad, with the war. There was no clean water. There were diseases, you see when children don’t drink clean water. Syrian mother of 4 children
3	In Syria they check for thalassemia and all… So, when they arrive here….. they should check them periodically, every 3 months… Syrian mother of 4 children
4	They are not allowed if they do not have the blood test (thalassemia) ….it does not have to do only with nieces and nephews, ….. it is better to have a blood test for thalassemia. Syrian/Palestinian mother of 12 children
5	We have no information… Little information about certain diseases……We also want to know, what kind of diseases are here in the Netherlands? How do we prevent that? What can we do about it? We do not know that. Syrian mother of 4 children
6	At school, my son… He drank lots of water. So at school they noticed and they’ve asked me… ‘We want to do an examination for him, we’re afraid of ……’. So they did a blood test, but luckily it was all good. Mother from Syria
7	In …….., they have told me to go to the doctor for a check-up. I just went to bed, I refused. One more time, I refused. The third time, they brought me themselves. And that has to do with… I have a lot of stress. I don’t want to talk to people about it, but I’ve been through a lot of things in Libya. So if I have to go somewhere, I get stressed. Then I think back to what I experienced in Libya. So, I cannot do it alone’. 16-year-old boy from Eritrea
8	We are not migrants, we are refugees. We suffered a lot. Our children come from a war zone. They suffered a lot. So our children need to be screened for PTSS and from there for other psychological issues. Syrian father of 7 children
9	My boy, a few years ago the resident status was refused, and then suddenly at 6 o’çlock in the morning the police was in his room, here in the reception centre. My daughter was so scared she peed on herself and my son…..look here… they knew he had psychological problems already….. it became worse…. And then he was brought to a detention centre…. Father from Iraq with two children
10	Our children have come here and suddenly they live in a different culture. Everything is allowed… It’s a different environment. So we notice that our children become a bit naughty, also against the parents. Father from Iraq with 2 children
11	“We are good parents. Don’t think that we didn’t raise them properly, we love our children. We must keep control over them. So if I grab my child’s hand like that, that doesn’t mean that I have to go to ‘Veilig Thuis’ (Centre for child abuse). You see, it’s different. And because things are different in the Netherlands, that does not mean that what we are doing it incorrect. And they [the Dutch] must understand that.” Father of 4 children, from Syria
12	An I-culture and a we-culture, that is the difference…… In school they learn to be independent. They have freedom. You exist for yourself. An I-culture. And they come from a we-culture. It is difficult to raise your child as me and only myself. Father from Syria with 7 children

Parents expressed worries about the health and nutritional status of their children because children suffered during the war, during the flight, and in the Netherlands (Table 3, quote 1–2). Some children had spent time in prison. Parents, especially mothers, and minors expressed needs and expectations regarding physical and mental health screening (Table 3, quote 8) and a more extensive initial health assessment or follow-up. Mothers expressed a wish to learn more about health, diseases, and prevention (Table 3, quote 4).

Parents from countries where thalassemia screening was obligatory before marriage, due to the high prevalence in the general population (Syria, Turkey), expected such screening in the Netherlands as well (Table 3, quote 3). UMs stated they would prefer a screening upon entry instead of going to a doctor on their own initiative (Table 3, quote 7).

Psychosocial issues were mentioned in every FGD. Parents told about nightmares, bedwetting, and anxiety of their children (Table 4), who had recurrent thoughts of the war.

**Table 4:** Initial health assessment in the Netherlands: experiences, needs, and expectations of asylum-seeking parents and UMs

	**Experience**	**Needs**	**Expectations**
General	- Referred to assessment without knowing where and how not knowing if they were seen by a volunteer or a doctor - Assessment in Turkey as invited refugees - School as extra “screening/monitoring” eye		- To be explained why and guided toward assessment - To have more than one assessment, e.g., follow-up after 6 months
Initial health assessment: physical health	They shared their worries: - Worries about health status of their child or themselves - Worries about screening for thalassemia - Worries about infectious diseases - Worries about nutrition status of the child	- To know the health status - To be informed about the screening for thalassemia as they are used to in their home countries - Information on diseases	- Address worries about health status and themselves by further investigations - Including hemoglobinopathies like thalassemia, infectious diseases, and micronutrient deficiencies - Address worries about nutrition and health status of the child
Initial health assessment: psychological health	They shared worries about the following items: - Nightmares - Bedwetting - Anxiety - Uncertainty about the future - Stress - Thoughts about the war	- To know the psychological status - To have access to psychological care - Information on psychological health - Continuity of care	- Psychological screening and support upon entry

They mentioned specific needs as refugees who fled from a war zone (Table 3, quote 8) and suffered severe psychological consequences (Table 3, quote 9). Children taken out of their beds at night by the police in the Netherlands suffered from severe post-traumatic stress symptoms. The parents and minors who traveled through Libya were especially worried about mental and trauma health issues. They were not able to talk about their experiences because it was too stressful (Table 3, quote 7).

Uncertainty about their residence status was a big stressor for the unaccompanied minors and influenced the ability to perceive health needs and act accordingly. Continuity of care for extensive mental health and psychosocial problems was discussed in all focus groups. Minors identified that they had major mental health problems, but they did not search for care nor were referred care or support. In all FGDs, unmet needs regarding mental health and social problems were mentioned.

### Access to health care

Differences between the health care system in the Netherlands and the country of origin were discussed (Table 5).

**Table 5:** Experiences, needs, and expectations of asylum-seeking parents and UMs on the Dutch health care

	**Experiences**	**Needs**	**Expectations**
Differences between health care in the Netherlands and the country of origin	- Use of antibiotics - Use of paracetamol and water - Referral system - Insufficient consulting hours - Waiting time - Access to specialized care - Postponed care due to relocations	- Information on health care system - Information on vaccinations - Information in general on health and disease	- Understanding the differences in health systems
Access to care	- Long waiting times - Difficulties with the referrals - Insufficient consulting hours - Difficulty navigating through the health care system - Postponed care due to relocations - Trust in the health care provider - Knowledge	- Information provision regarding health system	- Understanding how to navigate in the system to access the needed care
Information provision	- Information on health and diseases - Information on health care system - Information on vaccinations - Information in general	- More detailed information provision	- Understanding of health and disease and the health care system

None of the participants could explain the organization of the Dutch health care for asylum-seekers, nor understood the distinction between preventive Youth Health Care, primary care by a general physician, and secondary curative services. The need for information and education on health and diseases was discussed extensively, among refugees and among healthcare professionals. Various ideas and suggestions were brought up regarding factors that may influence the ability of refugees to obtain information, such as access to the internet, recall of diagnosis or care used in the country of origin, and social contacts in the neighborhood.

Participants perceived that treatment or care for refugees was postponed and delayed, and they expressed their concern about long waiting times and the complex Dutch referral system. Parents of children with complex health needs were often not informed in their own language about the condition of their child, or only with the help of an informal “interpreter,” for example, a relative with little understanding of the Dutch or English language.

Parents and minors had to get used to the reluctance of health care providers to subscribe antibiotics in the Netherlands, in comparison to receiving antibiotics over the counter in their country of origin.

The health professionals’ experiences with access to care for asylum-seeking parents and UMs, were related to the five access dimensions of Levesque (Table 6).

**Table 6:** Perceptions of health care professionals on access of care in terms of the five dimensions of access

**Access dimensions**^**a**^	**Subcategories**	***N 6***	**Example or quote **
Approachability	Poor health literacy	4	“They don’t always know. Sometimes I have to explain it a lot.”
	Contradicting health beliefs	2	“In their home country, they are used to always leaving with a pill. Well, that is very difficult for general practitioners to explain that that doesn’t help.”
	Lack of knowledge about health care rights	1	“They sometimes do not know that the care is free and also the follow-up care that comes with it.”
Acceptability	Cultural differences	3	“I notice that the cultural aspect is sometimes difficult.’ because it is very different than the Dutch culture.”
	Language difficulties	1	“They remain a vulnerable group. That you cannot express yourself properly, is just more difficult.”
Availability	Legal restrictions disallow treatments	2	“They must first have a status to be entitled to certain provisions.”
	Understaffing	2	“You sometimes notice that you have too few staff.”
	Insufficient consultation hours	2	“What I really encounter, I don't have enough time.”
	Time taken away from other patients’ consultation	1	“But what I do is also the nurse’s consultation hours. So, I am constantly disturbed.”
	Transportation difficulties	1	“Not at all so obvious that the other person has a car and can reach us, that they know the way with public transport well.”
Affordability	Medication costs	1	“If you have to take it chronically and you have such a budget, then it really adds up.”
Appropriateness	Poor communication between services	3	“Communication is poor between the COA and the Public Health Services”
	Poor transfer of medical files	2	“I get no report from anyone. I thought, where do I start?”
	Postponed care due to relocations	1	“At the time, there was contact with another hospital about it. But, the child dropped out of care due to relocations.”
Suggestions to improve care	More consultation hours	3	“We can improve access by having more consultation hours.”
	More experienced staff	2	“There just needs to be one more doctor, another day, and a nurse with more experience in screening.”
	More education for UMAs on healthy lifestyle	2	“I think you have to invest very intensively on healthy foods, smoking and alcohol.”
	Cooperation with a pedagogue	1	“Someone like a pedagogue should be present.”
	Cooperation with a pediatrician	1	“More cooperation with a paediatrician.”

*N* the number of interviewees who have mentioned the subcategories

^a^Dimensions of access to care by Levesque et al. [14] linked to the subcategories

The doctor-patient relationship was a recurrent topic, as it takes time to build such a relationship, especially in an intercultural setting. Professionals acknowledged that it took time to build trust and gain authority with the refugee population. Health care professionals underlined the importance of professional interpreters.

### Parenting in between cultures

Although not asked for explicitly, cultural change was mentioned spontaneously as part of parental and children’s well-being. Parents told they tried to maintain their own cultural values and practices, while their children quickly accommodated Dutch cultural practices at school. Parents perceived that this cultural gap led to uncertainty about norms and values, to parent–child conflicts (Table 3, quote 10 and 11), or to professionals not taking into account or respecting parental norms and values (Table 3, quote 12). Parents experienced difficulties in raising their children without their extended family and compatriots around.

Parents stated that their children had little social contact with other children. Relocations from center to center negatively influenced the establishment of a social network. Parents expressed their need for parental support (Table 3, quotes 10–12).

## Discussion

We explored the experiences of asylum-seeking parents and unaccompanied minors toward the initial health assessment of children upon entry and their perceptions toward access to health care in the Netherlands.

Parents and minors were not always aware of the scope and possibilities of the initial health assessment nor recalled to have had such an assessment. They were satisfied with the vaccination program but missed screening for specific diseases and for psychosocial problems. They expressed the need for an extended initial health assessment about the health and nutritional status of their children or themselves as minors, especially those who had been in a war zone for a long time or traveled through Libya. Their needs for trauma detection, support, or care were not met, and parents and minors expressed their wish for trauma support. Parents mentioned the importance of support for dealing with cultural transitions while raising their children. They experienced multiple barriers in access to care, which were corroborated by health professionals.

Parents and minors in our study were often unfamiliar with the initial health assessment, which is in line with a Swedish study, in which new migrants did not understand the rationale for screening, as they may have symptom-driven health-seeking behaviors [19]. In our study, lack of information on the health care system of the new country may have acted as a barrier in perceiving one’s own health needs and engaging in this system.

Our study participants experienced a mismatch between their health needs and expectations versus the initial health assessment. However, a more extensive health assessment does not necessarily lead to higher satisfaction because asylum-seekers may fear lack of confidentiality of the test results, which might influence the asylum procedure [19].

Parents realized they did not have access to standard screening for thalassemia in the Netherlands, a test obligatory before getting married in home countries with a high prevalence of thalassemia and other genetic blood disorders [20, 21]. Parents observed a gap between the neonatal screening in the Netherlands and the premarital screening in their home countries. As screening is not available to them in the Netherlands, this hindered their ability to reach health services and take part in the system.

Parents and minors who traveled through Libya were worried about possible acquired sexually transmittable diseases. In terms of Levesque, we interpreted this as trauma influencing their ability to perceive and recognize health needs which, in turn, hindered them to engage in the health care system (see Fig. 1).

The Central Mediterranean Route, passing through Libya, is one of the most dangerous routes for migrants [22]. The prevalence of (sexual) violence is high. Many women reported a pregnancy during travel [22, 23]. UNICEF rose alarm in 2020 about the high risk of UMs amidst violence and chaos of the unrelenting conflict [24]. In our study, the minors did not want to reveal what exactly happened in Libya. The Tigrinya interpreter told us in person after the FGD that the minors probably indirectly meant sexual abuse. Sexual abuse increases health risks, and the initial health assessment could be an instrument linking them to care.

Trauma was a salient topic in the FGDs. Parents reported symptoms of PTSS among their children. For the minors, the main stressor was the asylum procedure itself, leaving them in despair after a negative asylum request. The stress of uncertainty, lack of perspective, and social support with frequent relocations hindered them to engage in the health care system. A recent review concluded that forced migration and a prolonged asylum procedure, in addition to the complexity of the acculturation process, can contribute to higher levels of psychopathology [25].

Stress, war trauma, post-traumatic stress, and mental health problems were major barriers in participants’ ability to perceive health needs, to seek the right (preventive) services, and to engage in the health care system. As conceptualized by Levesque, refugee parents’ and minors’ limited or unfacilitated abilities to perceive, seek, reach, and engage (in) health care, all played a role in the access.

We recommend an improved initial health assessment that recognizes and addresses the health needs of asylum-seeking children, both physical and mental. With regard to physical health, this should include standardized screening for most common problems according to region of origin. Regarding mental health, we recommend a standardized screening with validated and cultural sensitive screening instruments. Such an initial health assessment might detect health needs better and may lead to more adequate referral. Furthermore, an adaptive and reciprocal approach of health providers is essential for enhancing the engagement of asylum-seeking children with the health care system, leading to improved health outcomes for this vulnerable population.

## Strengths and limitations of the study

A strength of this study is the inclusion of perspectives of three different stakeholder groups on the initial health assessment and access to health care as triangulation: asylum-seekers, parents and minors from various regions of origin, and health care professionals.

A limitation of the study was the language barrier, which limited communication between researchers and refugees. Even though professional interpreters were present, they may have adjusted the interview questions and refugees’ responses. Another limitation is that refugees are in a vulnerable position, and fear of the outcome of the asylum procedure may have influenced their responses. Another limitation is the possibility of selection bias because recruitment was done in the reception centers, and we did not have insight in motivation to participate or not. We only interviewed male UMs with a rejected asylum request; their responses might have been biased because they may be less motivated to seek healthcare even when having complaints than UMs still in asylum procedure or with a residence permit. We only recruited parents and UMs from one reception center, which may have biased our results [26].

## Conclusion

The perspectives of refugee families and unaccompanied minors revealed opportunities to improve the experience of and access to health care of refugees entering the Netherlands.

Specifically a risk-specific screening and mental health assessment. Health literacy and adequate education about health, diseases, and the health system would help them engage to the system and more adequately address their health needs. Improving the initial health assessment could enable asylum-seeking parents and minors to better recognize their health needs, reach the right services, engage in the health care system, and find appropriate services.

### Supplementary Information

Below is the link to the electronic supplementary material.

Supplementary file1 (DOCX 39 KB)

## Data Availability

Datasets in the form of transcripts, without information that might be traceable to persons, can be accessed by direct contact with the corresponding author.

## Abbreviations

FGDs: Focus group discussions
UMs: Unaccompanied minor asylum-seekers

## References

[1] UNHCR/WFP. Global trends forced displacement in 2019 2022 [cited 2022 2022–5–21]. Available from: https://www.unhcr.org/data.html

[2] IND. Asylum trends December 2017 t/m 2021 Available from: https://ind.nl/nl/documenten/03-2022/atdecember2021hoofdrapport.pdf

[3] Baauw A, Rosiek S, Slattery B, Chinapaw M, van Hensbroek MB, van Goudoever JB, Kist-van HJ (2018). Pediatrician-experienced barriers in the medical care for refugee children in the Netherlands. Eur J Pediatr.

[4] Statistiek CBv. Dossier Asiel, migratie en integratie 2019: cbs; 2022 Available from: https://www.cbs.nl/nl-nl/dossier/dosier-asiel-migratie-en-integratie

[5] Yun K, Matheson J, Payton C, Scott KC, Stone BL, Song L (2015). Health profiles of newly arrived refugee children in the United States, 2006–2012. Am J Public Health.

[6] Hunter P (2016). The refugee crisis challenges national health care systems: countries accepting large numbers of refugees are struggling to meet their health care needs, which range from infectious to chronic diseases to mental illnesses. EMBO Rep.

[7] Harkensee C, Andrew R (2021) Health needs of accompanied refugee and asylum seeking children in a UK specialist clinic. Acta paediatrica (Oslo, Norway : 1992) 10.1111/apa.15861 33783882

[8] Baauw A, Kist-van Holthe J, Slattery B, Heymans M, Chinapaw M, van Goudoever H (2019). Health needs of refugee children identified on arrival in reception countries: a systematic review and meta-analysis. BMJ Paediatr Open.

[9] Goosen S, Hoebe CJPA, Waldhober Q, Kunst AE (2015). High HIV prevalence among asylum seekers who gave birth in the Netherlands: a nationwide study based on antenatal HIV tests. PLoS ONE.

[10] Hebebrand J, Anagnostopoulos D, Eliez S, Linse H, Pejovic-Milovancevic M, Klasen H (2016). A first assessment of the needs of young refugees arriving in Europe: what mental health professionals need to know. Eur Child Adolesc Psychiatry.

[11] Marquardt L, Kramer A, Fischer F, Prufer-Kramer L (2016). Health status and disease burden of unaccompanied asylum-seeking adolescents in Bielefeld, Germany: cross-sectional pilot study. Trop Med Int Health.

[12] GGDGHOR. Publieke Gezondheidszorg Asielzoekers (PGA): GGD-GHOR; 2022 Available from: https://ggdghor.nl/thema/publieke-gezondheid-asielzoekers

[13] Vermeulen G, Slinger K, Zonnenberg I, Drijfhout I, Appels R (2017). Asielzoekerskinderen en het Rijksvaccinatieprogramma (RVP). JGZ Tijdschrift voor jeugdgezondheidszorg.

[14] Levesque JF, Harris MF, Russell G (2013). Patient-centred access to health care: conceptualising access at the interface of health systems and populations. Int J Equity Health.

[15] Brandenberger J, Sontag K, Duchene-Lacroix C, Jaeger FN, Peterhans B, Ritz N (2019). Perspective of asylum-seeking caregivers on the quality of care provided by a Swiss paediatric hospital: a qualitative study. BMJ Open.

[16] Dawson-Hahn E, Koceja L, Stein E, Farmer B, Grow HM, Saelens BE (2020). Perspectives of caregivers on the effects of migration on the nutrition, health and physical activity of their young children: a qualitative study with immigrant and refugee families. J Immigr Minor Health.

[17] Tong A, Sainsbury P, Craig J (2007). Consolidated criteria for reporting qualitative research (COREQ): a 32-item checklist for interviews and focus groups. Int J Qual Health Care.

[18] Braun V, Clarke V (2006). Using thematic analysis in psychology. Qual Res Psychol.

[19] Nkulu Kalengayi FK, Hurtig AK, Nordstrand A (2015). Perspectives and experiences of new migrants on health screening in Sweden. BMC Health Serv Res.

[20] Kadhim KA, Baldawi KH, Lami FH (2017). Prevalence, incidence, trend, and complications of thalassemia in Iraq. Hemoglobin.

[21] Vichinsky E, Hurst D, Earles A, Kleman K, Lubin B (1988). Newborn screening for sickle cell disease: effect on mortality. Pediatrics.

[22] Reques L, Aranda-Fernandez E, Rolland C, Grippon A, Fallet N, Reboul C (2020). Episodes of violence suffered by migrants transiting through Libya: a cross-sectional study in “Medecins du Monde’s” reception and healthcare centre in Seine-Saint-Denis. France Confl Health.

[23] Argent E, Emder P, Monagle P, Mowat D, Petterson T, Russell S (2012). Australian paediatric surveillance unit study of haemoglobinopathies in australian children. J Paediatr Child Health.

[24] Fore H. Tens of thousands of children at risk amidst violence and chaos of unrelenting conflict: Unicef; 2020 Available from: https://www.unicef.org/press-releases/libya-tens-thousands-children-risk-amidst-violence-and-chaos-unrelenting-conflict

[25] Pluck F, Ettema R, Vermetten E (2022). Threats and interventions on wellbeing in asylum seekers in the Netherlands: a scoping review. Front Psychiatry.

[26] Braun V, Clarke V (2021). To saturate or not to saturate? Questioning data saturation as a useful concept for thematic analysis and sample-size rationales. Qualitative Research in Sport, Exercise and Health.

