# Supplementary material for: Perspectives of refugee parents and unaccompanied minors on initial health assessment and access to care
Source: Eur J Pediatr. 2024 Apr 9;183(7):2871–80. doi: 10.1007/s00431-024-05523-5 (PMC11192806; doi:10.1007/s00431-024-05523-5)
Supplement: Supplementary file 1 — Supplementary file1 (DOCX 39 KB) [file 431_2024_5523_MOESM1_ESM.docx]

***Appendix I: Statistics: Asylum requests in the Netherlands 2015-2021 and children (0-18years)***

|  | 2015 | 2016 | 2017 | 2018 | 2019 | 2020 | 2021 | Total |
| --- | --- | --- | --- | --- | --- | --- | --- | --- |
| Total asylum requests | 58,880 | 31,642 | 31,327 | 30,380 | 29,435 | 19,132 | 36,620 | 237,416 |
| asylum requests < 18 years | 10,595 | 6,240 | 4,290 | 5,520 | 5,690 | 3,190 | 6,240 | 41,765 |
| asylum requests unaccompanied minors | 3,859 | 1,707 | 1,181 | 1,225 | 1,046 | 986 | 2,191 | 12,195 |
| Family reunification | 13845 | 11,814 | 14,490 | 6,463 | 4,179 | 3,863 | 10,120 | 64,774 |

*Source:* ^1^, ^2^

***Appendix II: Topic guide for Focus Group Discussions***

**Theme I: Expectations, needs and experiences with health care in the Netherlands**

1. Initial health assessment by the Youth Health Department within the Public Health services

- What do you remember from the first consultation at the Youth Health Doctor of your child in the Netherlands, for example when your child received their vaccination
- Were you present? When did it take place?
- What were the topics that were discussed and were did the doctor look at?
- What information was given about the initial health assessment?
- Do you remember which vaccinations were given?

1. What were your positive and your less positive experiences during your contact with the Youth health doctor?

- For example: language, provision of information, vaccinations, follow-up etc

1. What were your expectations from the Dutch health system (for your child)

- What did you expect from the health care in the asylum seekers centres
- What vaccinations did you expect
- What did you expect from the health care provision during pregnancy and delivery and care for the new-born
- Which tests did you expect for diseases that are common in your country of origin
- What did you expect for health care provision for mental health

1. When a child is born in the Netherlands, they will receive antenatal care as well as neonatal screening for diseases like thalassemia and sickle cell disease. This is preventive care.

- How is this arranged in your country of origin? What are the differences in preventive services between your country of origin and the Netherlands?
- What is your opinion and what is your expectation on this difference?

1. In your country of origin certain condition are more prevalent. We do not test for this conditions when your child/you arrive in the Netherlands.

- What is your opinion on this?

1. Children receive, if necessary, additional vaccinations. If it would be possible to test refugee children for the most common disease s with a preventive purpose, what is your opinion on this?

- What would be the advantages?
- What would be the disadvantages?
- When it would be offered on voluntary basis, would you join the program?

1. The intake consists of an anamnesis, additional vaccinations and if needed, a referral. How would, the initial health assessment in the asylum seekers centres by the youth health department look like?

**Theme II:**  **Perceived access to health care services in the Netherlands**

1. What is your experience with access to health care?

- Where do you go when you child is ill?
- Do you know how to make an appointment with the general practitioner?

1. What were your expectations from the Dutch health care system? Is this what you expected? What are the differences?
2. What is the difference between the accessibility of the health care system between your country of origin and the Netherlands?
3. Are you satisfied with the access to health care in the Netherlands?

- If not, what are the problems you encounter?
- For example: transfers from one asylum sekkers centre to the other, medical history, medical files, communication with your doctor, provision of information, cultural differences

1. What do you need to receive optimal care for your child/ you?

**Theme III: Topic Guide of the Interviews of Health Care Professionals**

Theme Questions

Introduction - Purpose and structure of the interview

Audio recording and anonymity

General

a. How long have you been working with refugee children and what is your role?

b. What are the countries of origin of the children you work with?

c. Approximately how many refugee children do you see per month?

Initial health assessment

- Brief explanation of preventive healthcare and the role of JGZ

a. What do you think of the current newcomer survey (history and vaccinations)?

- The diseases for which vaccinations are administered

- Attention to psychological health.

- Information provision

- Completeness/comprehensiveness.

- Do you think that the newcomer survey meets the health needs of refugee children?

b. Refugee children have different health needs compared to Dutch children in terms of health care.

- What in your experience are the needs of refugee children?

c. In country of origin of asylum-seekers different diseases occur than here in the Netherlands.

(e.g. anemia, hemoglobinopathies, hepatitis B and C, HIV, (latent)

tuberculosis, parasitic infections). Refugee children are not preventively tested upon entry into the Netherlands. What is your opinion about this?

d. What would you think about an initial health assessment for asylum-seeking children upon entry in the Netherlands, for common diseases depending on the country of origin?

- Advantages disadvantages?

e. What should be included in the initial health assessment by a youth nurse / youth doctor in the Netherlands?

Accessibility to healthcare

Availability - Ability to perceive

Acceptability - Ability to seek

Availability & Accommodation - Ability to reach

Affordability - Ability to pay

Appropriateness - Ability to engage

a. What knowledge do you have about the background of your patients?

b. What do refugee parents/children/UMs know about the health systems and their rights to health care?

c. Are there accessibility barriers related to the care you provide?

d. To what extent do you experience that language, culture, and religion influence care?

How do you think this can be improved?

e. What could you tell us about accessibility of care for refugees (transport/language)? What kind of support is provided?

f. To what extent are your patients aware of their health insurance and possible excess amount to receive the necessary care?

g. To what extent does it happen that patients have to pay additional for transport, medication or

additional medical assistance?

h. Do you feel that your patients understand you sufficiently during the consultation? If so, how do you act until they understand you? If not so, what is the reason for this?

Closing/Acknowledgements

i. Do you experience any further barriers to provide optimal care to refugee children?

j. What would be needed to improve (accessibility) care for refugee children?

***Appendix III: Coding scheme***

**Theme I : Expectations, needs and experiences with health care in the Netherlands**

1. Barriers

Distrust in caregiver competence

Referral difficulties to connect with health services

Health professional is not present

Long waiting time to connect with health care services

Postponed care due to delayed transfer of medical files

Perceived insufficient time of GP consults of GZA

Lack of support from GZA

Lack of appropriate care

Lack of health literacy

Difficulty navigating through healthcare system

1. Expectations

The GP concept

The needed authorization of a health professional to access medicine

Antibiotics are not readily available/prescribed

Restrictions to access hospital care

1. Perceived needs

Perceived need for health screening due to war trauma

Perceived need for blood test and screening upon arrival in the Netherlands

Education on raising children in the Netherlands

Perceived need periodic physical examination

Perceived need for mental health care

Perceived for education on disease prevention

1. Experienced stressors

Aversion to vaccinations

AZC accommodation reinforces stress

Legal restrictions reinforce stress

Police invasion reinforces stress

1. Positive experiences

Good care at child birth

Good screening

Good caregiver support from health care services

**Theme II:**  **Perceived access to health care services in the Netherlands**

| **Health systems** |  | **Abilities of the asylumseekers** |
| --- | --- | --- |
| **1 Approachbility** |  | **Ability to perceive**  Health literacy  Health believes  Awareness of one’s right to health care |
| **2 Acceptability**  Availability and acceptability: increased time requirement |  | **Ability to seek**  Knowledge of language  Cultural differences |
| **3 Availability**  Time taken away from other patients consultation  Increased time requirement  Relocations limit access to care |  | **Ability to reach**  Access to treatment knowledge of legal restrictions |
| **4 Affordability**  Costs medication  Transportation to hospital |  | **Ability to pay**  Access to extra funds |
| **5 Appropriateness**  experienced staff shortage by HP  relocations limit access to care  missing / late transfers of medical files  lack of communication between healthcare services |  | **Ability to engage**  Caregiver support |

***Appendix IV: Characteristics of parents***

| ***Characteristics of parents of asylum-seeking children and UMs for the quotes in table 3 and 5*** | | | |
| --- | --- | --- | --- |
| Ref | Mentioned in FGD | Page and line | Characteristics of participant |
| 1 | 1 | 1.9 line 309 | Syrian father with 4 children 1months, 6,10 and 12 years. Since 11 months in the Netherlands. |
| 2 | 4 | 4.5 line | Syrian mother of 4 children, 2 in the Netherlands aged 16 and 18 years and 2 children in Syria. Unknown how long in the Netherlands. |
| 3 | 4 | 4.11 | Syrian mother of 4 children, 2 in the Netherlands aged 16 and 18 years and 2 children living in Syria. |
| 4 | 2 | 2.6 -2.7 | Syrian/Palestinian mother of 12 children. Since 9 months in the Netherlands. |
| 5 | 4 | 4.11 line | Syrian mother of 4 children, 2 in the Netherlands (16 and 18 years) and 2 in Syria. |
| 6 | 2 | 2.6 line | Mother from Syria. Not known how many children and not known how long in the Netherlands. |
| 7 | 5 | 1.9 line | 16-year-old boy from Eritrea. Since 7 months in the Netherlands. |
| 8 | 1 | 1.10 line 343 | Syrian father of 7 children in the ages of 2-15 years old. Since 1 year and 3 months in the Netherlands |
| 9 | 1 | 1.5 line 163 | Father from Iraq with two children aged 9 and 13. Since 3 years in the Netherlands. |
| 10 | 1 | 1.10 line 374 | Father from Iraq with 2 children 9 and 13. Since 3 years in the Netherlands |
| 11 | 1 | 1.11 lone 407 | Father from Syria with 4 children aged 9 months, 3, 12 and 13 years. Since 1 year and 3 months in the Netherlands. |
| 12 | 1 | 1.12 line 429 | Father from Syria with 7 children aged 2 to 15 years of age, not specified. Not known how long in the Netherlands. |

|  | 2015 | 2016 | 2017 | 2018 | 2019 | 2020 | 2021 | Total |
| --- | --- | --- | --- | --- | --- | --- | --- | --- |
| Total asylum requests | 58,880 | 31,642 | 31,327 | 30,380 | 29,435 | 19,132 | 36,620 | 237,416 |
| asylum requests < 18 years | 10,595 | 6,240 | 4,290 | 5,520 | 5,690 | 3,190 | 6,240 | 41,765 |
| asylum requests unaccompanied minors | 3,859 | 1,707 | 1,181 | 1,225 | 1,046 | 986 | 2,191 | 12,195 |
| Family reunification | 13845 | 11,814 | 14,490 | 6,463 | 4,179 | 3,863 | 10,120 | 64,774 |
